# Pupil‐Adaptive Retina Projection Augment Reality Displays With Switchable Ultra‐Dense Viewpoints

**DOI:** 10.1002/advs.202416961

**Published:** 2025-03-17

**Authors:** Haonan Jiang, Yuechu Cheng, Zhibo Sun, Zhengnan Yuan, Huajian Jin, Yipeng Huo, Man‐Chun Tseng, Fion Yeung, Hoi‐Sing Kwok, Enguo Chen

**Affiliations:** ^1^ National & Local United Engineering Laboratory of Flat Panel Display Technology Fuzhou University 2 Xueyuan Road Fuzhou Fujian Province 350108 China; ^2^ State Key Laboratory of Advanced Displays and Optoelectronics Technologies and Center for Display Research Department of Electronic and Computer Engineering The Hong Kong University of Science and Technology Clear Water Bay Kowloon Hong Kong 999077 China

**Keywords:** augmented reality, multi‐viewpoints display, photo‐alignment liquid crystal dammann grating (p‐LCDG), retina projection display(RPD)

## Abstract

Multi‐viewpoint retina projection displays (RPD) with an expanded visible range have been utilized in recent augmented reality (AR) systems to address the vergence‐accommodation conflict (VAC) by providing a long depth of field (DOF). However, these fixed multi‐viewpoint RPD systems still face a common critical challenge of imaging overlap or discontinuity when eyes rotate or under varying ambient light. To address this, an RPD AR system featuring switchable ultra‐dense viewpoints is presented, enabled by a photo‐alignment liquid crystal Dammann grating (p‐LCDG). The number of viewpoints reaches 49, which forms an ultra‐high density of diffraction lattice in front of the pupil with a record high rotation precision of 1.28°/viewpoint, allowing for a substantial range of 36 mm^2^. More importantly, the spacing of adjacent viewpoints is 0.532 mm, much smaller than the minimum radius of the pupil (≈1 mm). To facilitate viewpoint switching, a light selector is implemented, ensuring that only the light from a specific viewpoint reaches the eye, which effectively eliminates the image missing or discontinuity. By combining eye tracking technology, the viewer can consistently perceive a singular and clear image from the proposed RPD system, achieving seamless switching of viewpoints. This innovative design paves the way for high‐performance RPDs in AR applications.

## Graphical abstract

This paper presents a seamless retina projection AR display with 49 switchable ultra‐dense viewpoints, achieving a record high rotation precision of 1.28°/viewpoint. With a light selector, only a singular image can be consistently perceived by eye‐tracking without image missing or overlapping regardless of dynamic pupil under different ambient lights. This design paves the way for high‐performance RPDs in AR applications.

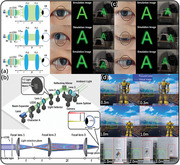

## 1 Introduction

In recent years, the advancement of emerging display technologies, including mini‐light‐emitting diode (LED),^[^ ^1^, ^2^, ^3^, ^4^ ^]^ micro‐LED,^[^ ^5^, ^6^, ^7^, ^8^, ^9^ ^]^ and quantum‐dot LED (QLED),^[^ ^10^, ^11^, ^12^, ^13^, ^14^, ^15^, ^16^ ^]^ has presented unprecedented opportunities to micro‐projection displays,^[^ ^17^, ^18^ ^]^ particularly in the realm of augmented reality/virtual reality (AR/VR). These display technologies offer promising solutions to enduring challenges in AR/VR devices, such as volume, brightness, power consumption, and resolution.^[^ ^19^ ^]^ Notably, AR displays present a more complex challenge than VR, as they necessitate the seamless integration of virtual and real images for user perception.^[^ ^20^ ^]^

Currently, there are two main types of AR display devices: AR head‐mounted displays (HMDs) and AR glasses. The former has been widely used in specific fields of military and aviation, providing high optical performance and wide field of view (FOV) exceeding 100°.^[^ ^21^, ^22^ ^]^ Additionally, with the use of a microlens array or a freeform lens group, three‐dimensional (3D) displays, and multifocal displays can be presented in front of the human eye.^[^ ^23^, ^24^ ^]^ However, achieving exceptional imaging necessitates sacrificing weight and volume, which are essential for everyday use in the civilian market. The development of AR glasses compensates for the shortcomings of HMDs.

Generally, AR glasses consist of a micro‐display projector and an imaging system. Based on different optical principles, AR glasses can be divided into four categories: geometric design, pin‐light, waveguide, and Maxwellian display.^[^ ^25^ ^]^ The geometric design is a traditional approach that uses a freeform half mirror or birdbath optics in its light combiner.^[^ ^26^, ^27^ ^]^ This type typically offers a wide FOV and excellent aberration correction, like HMDs. However, the bulky system may cause health problems when worn for extended periods. Apart from the traditional structure mentioned above, waveguides follow the principle of total internal reflection (TIR) within the glass. The eyebox can be expanded by coupling out at different positions of the waveguide, providing a good viewing experience even when the eyes rotate while maintaining a slim volume.

Nowadays, waveguide combiners primarily adopt diffractive gratings as couplers, including surface relief grating (SRG),^[^ ^28^ ^]^ volume holographic grating (VHG),^[^ ^29^ ^]^ and polarization volume grating (PVG).^[^ ^30^ ^]^ SRG consists of slanted micro‐structures with periods, which offer broad design freedom. VHG has high performance in single‐order diffraction and is simple to prepare, but its diffraction bandwidth is relatively narrow, limiting the FOV and efficiency. PVG, which combines Bragg grating and liquid crystal material, expands the diffraction bandwidth with high efficiency and can achieve high performance in true color by laminating the PVG.^[^ ^31^ ^]^ Among these mainstream waveguide combiners, the vergence‐accommodation conflict (VAC) issue is always a big challenge.

The emergence of Maxwellian displays, also known as retinal projection displays (RPDs), can inherently eliminate the VAC issue due to their long depth of field (DOF).^[^ ^32^ ^]^ This ensures that users can see all objects clearly without continually refocusing. However, the small aperture in RPDs results in a relatively small eyebox. When users rotate their eyes, the virtual image may no longer be visible. To overcome this challenge, current research primarily focuses on expanding the eyebox, including pupil steering or duplication (see Section S1, Supporting Information for their details). Both methods enlarge the eyebox and allow users to view AR images while their eyes are rotating. However, they have not addressed a critical issue. The position of multiple viewpoints is fixed, while the diameters of pupils dynamically change (up to 4–6 mm in a dark environment and down to 1–2 mm in a bright environment) under varying ambient lighting conditions(The detailed information about the effect on pupil vision with different pupils and different positions of viewpoint can be seen in Section S2, Supporting Information). Consequently, a specific type of pupil steering or duplication RPD may cause image loss or overlap in different environments, making it unsuitable for use in bright sunlight or nighttime conditions.

To address this issue, we present a novel RPD display using a photo‐alignment liquid crystal Dammann grating (p‐LCDG) to generate ultra‐dense 49 multi‐viewpoints with a 2D array, where the distance between adjacent multi‐viewpoints is significantly smaller than the extreme radius of the pupil. By incorporating a light selector, switchable multi‐viewpoints are simultaneously achieved with highly controllable rotation precision for eye tracking in AR displays. At any time, only the light from the selected multi‐viewpoint can pass through the system and be captured by the human pupil, effectively eliminating image overlap or discontinuity. This light‐controlled system allows virtual AR information to be freely observed with high rotation modulation precision full‐time, regardless of eye rotation or dynamic aperture.

## 2 Fundamental

**Figure 1:**
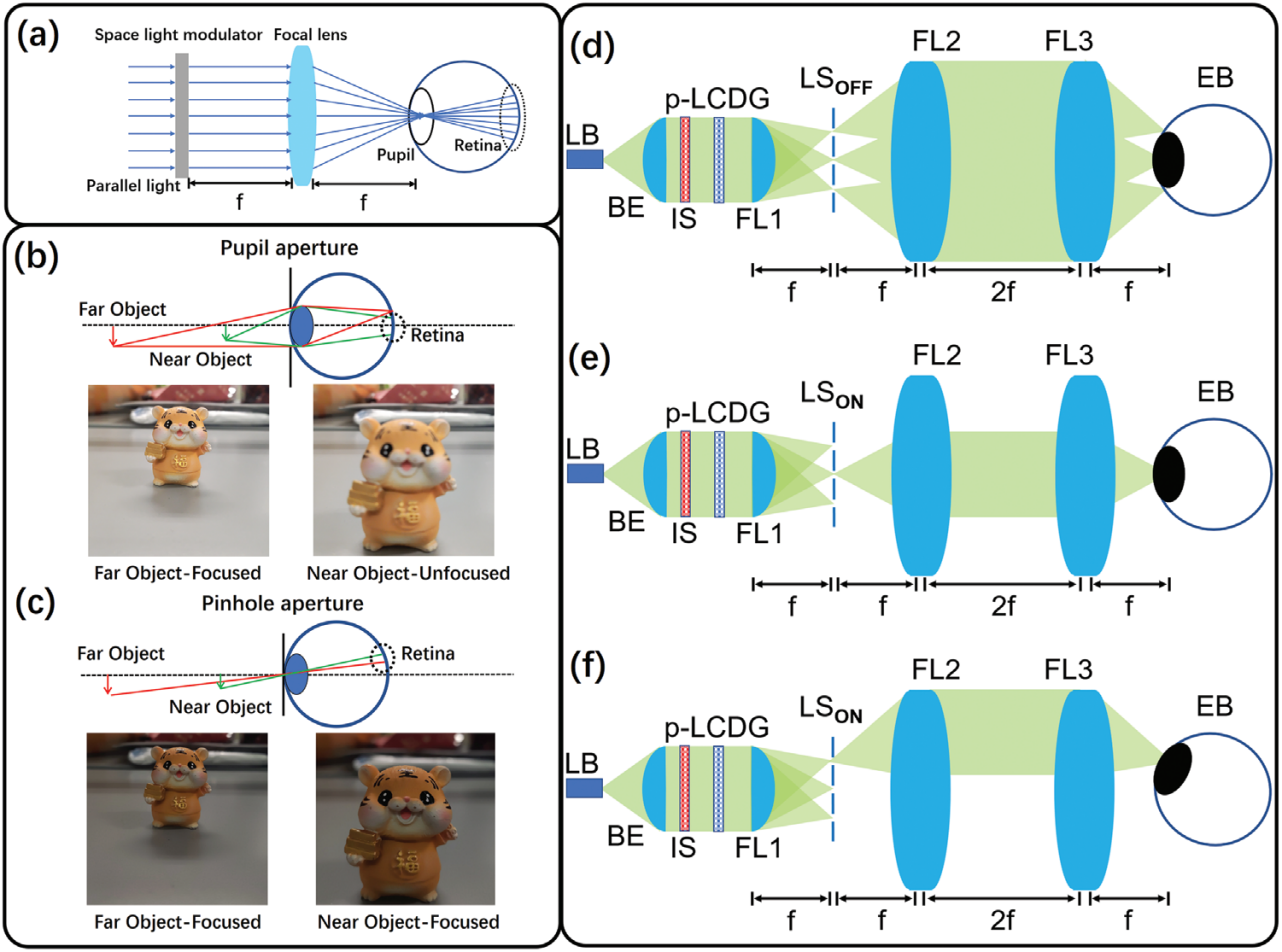
a) Fundamental principle of a typical AR RPD system. Comparison between b) the normal aperture and c) the pinhole case of the RPD system for the human eye. Schematic of the proposed AR RPD system when d) the light selector is off, e) the light selector is on for the central viewpoint, and f) the light selector is on for the edge viewpoint. (LB: Laser Beam. BE: Beam Expander. IS: Image Source. DG: Dammann Grating. LS: Light Selector. FL: Focal Lens. EB: Eyeball).

An AR display essentially consists of a projector equipped with a micro‐display panel and an eyepiece system. The original virtual image generated by the micro‐display is projected into a larger one that can be refocused by the pupil without affecting the real image.^[^ ^33^ ^]^ In contrast, the theory of the Maxwellian view considers the human eye as a component of the system.^[^ ^34^, ^35^, ^36^, ^37^ ^]^ In this system, the display plane and the human retina plane become a pair of conjugate object‐image planes. Instead of being refocused by the eyeball, it uses a spatial light modulator (SLM) to overlay image information at different aperture heights (h) and azimuthal angles (θ), corresponding to the receiving points on the retina, as illustrated in **Figure** **1**a. The FOV of this system is only related to the aperture of the light beam or the numerical aperture (NA) of the focal lens. Additionally, RPD can be regarded as a pinhole imaging model with an infinitesimal exit pupil diameter, as shown in Figure 1b,c.^[^ ^38^ ^]^ For an ideal pinhole imaging system, the DOF is infinite. As a result, the clarity of the image is not significantly affected by the changing position or tilt angle of the image plane, but only the magnification or distortion is influenced. With a sufficiently long DOF, vergence‐accommodation‐free imaging can be achieved by RPD, as the virtual image remains clear during the adjustment process of the human eye's focal length.^[^ ^39^ ^]^ Although RPD is not an ideal pinhole imaging model, it still holds distinct advantages of providing clearer images, higher contrast, and a longer DOF compared with traditional AR imaging systems.

The proposed RPD for AR displays combines a laser light source with a p‐LCDG and a 4f system.^[^ ^40^, ^41^, ^42^ ^]^ The fundamental principle of the proposed AR system is illustrated in Figure 1d–f. First, the green laser light is expanded by a beam expander to form parallel light with a certain aperture. Afterward, the light is diffracted by the p‐LCDG and then passes through the focal lens to form a multi‐viewpoint matrix on the focal plane. Here, a light selector is set to determine which viewpoint can pass through based on the pupil's position. Finally, the light in the selected position can be refocused on the pupil plane by the 4f system. To maintain the same magnification throughout the system, the same focal length of FL1 is kept as those in the 4f system. When the light selector is closed, the diffraction matrix forms in front of the pupil, as depicted in Figure 1d. When the light selector is active, only the central viewpoint will open for the light if the human eye is located straight ahead (shown in Figure 1e). If the human eye moves to the edge of the diffraction matrix, the light selector allows only the light at the edge viewpoint to pass through (shown in Figure 1f). In this way, the 2D viewpoint array generated by the p‐LCDG can be controlled by the light selector, and only one viewpoint's light can be captured by the human eye. This allows the human eye to rotate freely within a certain range and keep the virtual image centered in the pupil at all times. With this mechanism, the problem of image overlap or discontinuity can be resolved. Moreover, users can always see the clearest virtual image within a certain range of multi‐viewpoints due to foveated imaging.^[^ ^43^, ^44^ ^]^

## 3 Architecture and Simulation

### 3.1 System Modeling

**Figure 2:**
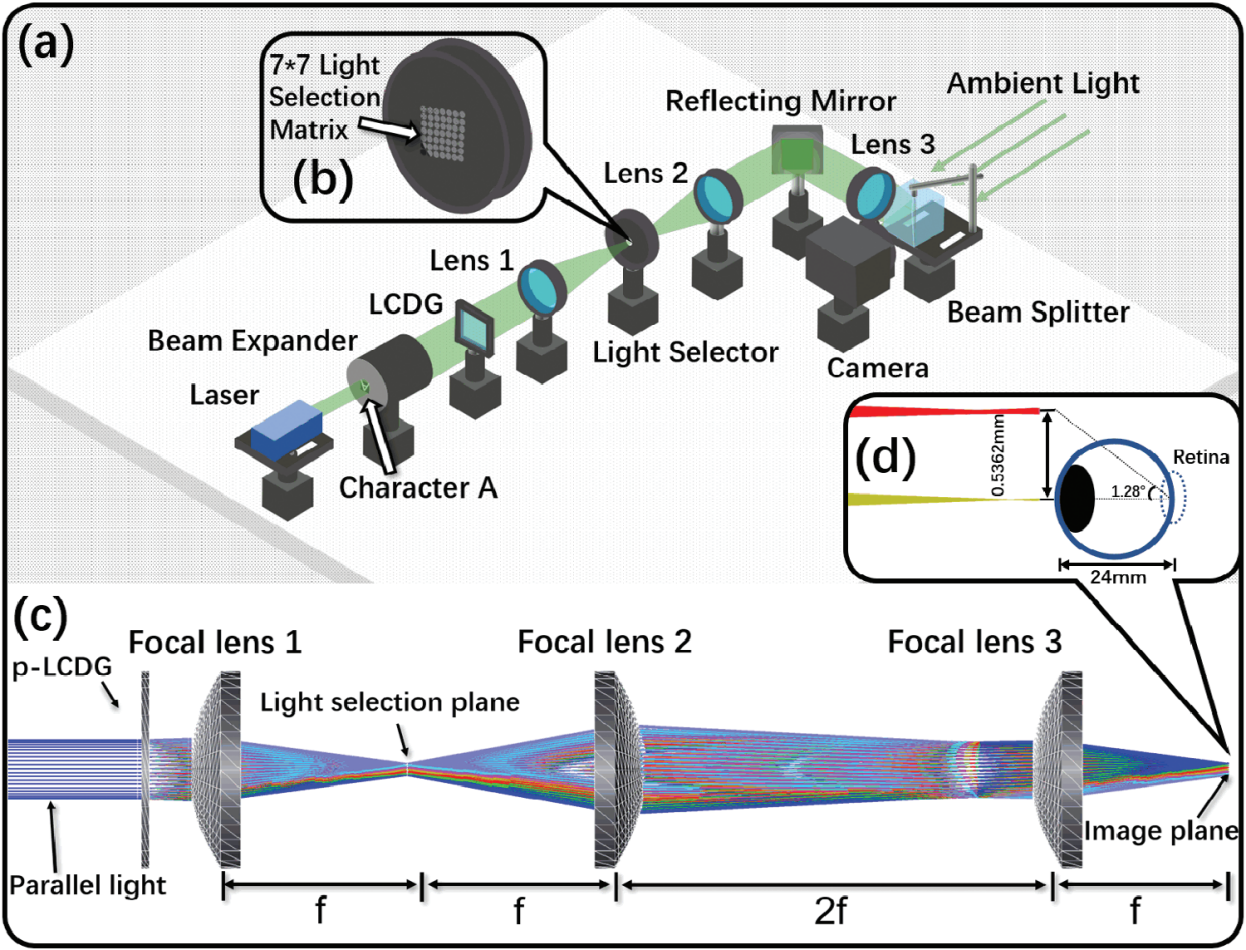
a) Schematic of the system modeling of the proposed AR RPD system with switchable multi‐viewpoints, including b) a 7 × 7 light selection matrix. c) Tw2D layout of the system. d) The measured distance between the viewpoints is highly consistent with the theoretical value.

The entire switchable multi‐viewpoint AR RPD system is built using a p‐LCDG and a 4f system, with its 3D model depicted in **Figure** **2**a. For the light source, a monochromatic green laser with a wavelength of 532 nm is used. A light modulator showing a character “A” is used to produce a virtual image. A p‐LCDG with 7 × 7 spots is chosen to achieve a balanced display effect in each viewpoint. Additionally, the light selector (Figure 2b) is positioned at the focal plane between Lens 1 and Lens 2, featuring a 7 × 7 matrix with small mechanical apertures that determine whether the light passes through or not. After Lens 2, the light path is folded by a reflecting mirror, creating an “L” turn. In front of the camera (simulating the human eye), the ambient object and virtual image are overlaid by a beam splitter. Here, the camera lens is positioned at the RPD's focal plane.

### 3.2 Optical Simulation

The RPD's optical performance is simulated by setting three focal lenses as sequential elements and the p‐LCDG as a non‐sequential component. The light wavelength is set to monochromatic green (λ = 532 nm), and the grating period is set to 50 µm. As shown in Figure 2c, the simulation begins with parallel light from a beam expander. The grating contains two elements, one with a horizontal direction and the other with a vertical direction, to simulate the corresponding 45° diffraction direction in the p‐LCDG. For the lens group, we choose 3 commercial aspherical lenses from *Edmund optics* with identical focal length to maintain the same magnification in the whole system (see the detailed optical parameters of the lens in Section S3, Supporting Information). Among them, Focal lens 1 is a focal lens, and Focal lenses 2 and 3 are Fourier lenses for the 4f system. The light diameter is set to 15 mm, and the light selector is replaced by two air optical spaces. As illustrated in Figure 2d, the spot distance on the image plane is manually measured to be 0.5362 mm, which is almost equal to the theoretical value of 0.532 mm. Here, on one hand, the spot distance of 0.532 mm is set to be smaller than the minimum radius of the human pupil (≈1 mm), which is adaptive to use under strong ambient light. On the other hand, the range of the diffraction lattice (≈36 mm^2^) is large enough to cover the maximum radius of the pupil (≈2 mm) under weak ambient light. This kind of pupil adaptation will help people use AR RPD during the whole day. Additionally, the rotation angle between two viewpoints for the eyeball is calculated to be 1.28°, where the rotation precision is equal to 1.28°/viewpoint. Here, the distance of the light spot can be obtained using the following Equation (1):

(1) a=f×tanθ

where a is the diffraction angle, θ is the distance of the light spot, and f represents the focal length.

**Figure 3:**
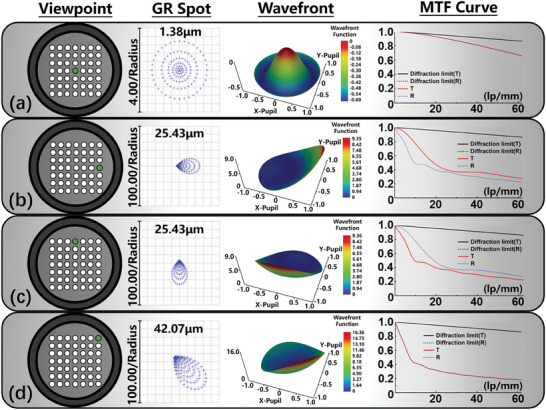
Optical analysis results for 4 typical viewpoints including a) center, b) edge of horizontal diffraction, c) edge of vertical diffraction, and d) edge of 45° diffraction. The results include the GR spot on the focal plane of the light selector, and the wavefront and MTF performance on the image plane.

The geometric radius (GR) on the focal plane of the light selector is analyzed to determine the appropriate aperture size for each viewpoint. Only the central and edge viewpoints are shown in **Figure** **3**a–d, and the entire 10 viewpoints analysis can be seen in Section S4 (Supporting Information). The GR of the central viewpoint (Figure 3a) is only 1.38 µm, while the other viewpoints at the edge of horizontal, vertical, and 45° directions are several times larger than that of the central viewpoint (Figure 3b–d). Among them, the edge viewpoint in the 45° direction is the farthest from the central area, and its GR reaches 42.07 µm (Figure 3d), complying with the aberration law of diffractive optical elements. After exploring the light spot of each viewpoint on the focal plane of the light selector, the size of the mechanical aperture on the light selector can be determined, which should be larger than 84 µm and smaller than half of the distance among each diffraction spot (≈250 µm).

Then, the image quality on the image plane is analyzed. From the wavefront plot, it can be seen a similar aberration trend from the central to the marginal viewpoint. The error peak in the central viewpoint is ≈−0.5, while the 45° edge reaches 16, which indicates the image quality deteriorates sharply as the diffraction distance increases. In addition, the modulation transfer function (MTF) performance on the image plane is also analyzed for the system. As shown in Figure 3a, the MTF of the central viewpoint is greater than 0.7 at the Nyquist frequency. Here, the display resolution is set to 1920 × 1080, and the size of the single unit of the display panel is set to 8 µm (equal to the cut‐off frequency of 62.5 lp mm^−1^). The MTF at the edge of the vertical and horizontal directions is greater than 0.3 (Figure 3b,c), while the MTF of the edge viewpoint at the 45° direction is greater than 0.2 (Figure 3d), which is satisfied with the minimum resolution baseline in AR display.^[^ ^45^, ^46^ ^]^ The image quality of the diffraction matrix at the off‐axis is not as good as that at the on‐axis because of the optical path difference, which could be further improved by the meta lens or other new devices.^[^ ^47^, ^48^, ^49^, ^50^ ^]^

The effect of different optical parameters on the entire optical system is systematically analyzed, and the results can be found in Section S5 (Supporting Information). Considering the preparation condition, the following optical parameters of the p‐LCDG are finally determined: lens focal length of 50 mm, grating period of 50 µm, light aperture of 15 mm, and FOV of 17.1°. The corresponding eyebox is 6 mm × 6 mm (The calculation of eyebox size can be seen in Section S6 (Supporting Information), and the three focal lenses use a commercially available one with a 10th‐order aspherical surface.

## 4 Experimental Section

### Preparation and Characterization

**Figure 4:**
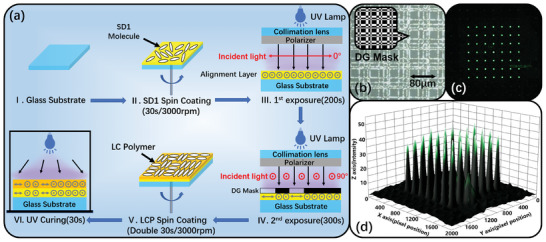
a) The preparation procedures of the proposed p‐LCDG. b) SEM image for the p‐LCDG's diffraction state. c) Diffraction lattice with the monochromatic green laser light source (λ = 532 nm). d) The diffraction intensity of 49 viewpoints was detected by the light sensor.

The p‐LCDG was a kind of binary phase grating (0, π) with special aperture functions, which produces a certain number of equal‐intensity light spots because of the Fraunhofer diffraction. To obtain the complete structure of grating, the phase distribution of the normalized period needs to be provided first, with the details found in Section S7 (Supporting Information). With the normalized period, the corresponding mask can be fabricated by copying the structure of the normalized period to be a specific size we want, as shown in **Figure** **4**a. Here, the p‐LCDG was prepared by patterned photo‐alignment due to the advantages of a simple fabrication process, cheap cost, and high structure resolution.^[^ ^51^, ^52^ ^]^ The preparation procedures of p‐LCDG are depicted in Figure 4a‐I–VI, which mainly include a spin coating for the photo‐alignment material and liquid crystal polymer (LCP) and a two‐step exposure to generate the two domains of the Dammann grating (DG) phase profile.^[^ ^53^ ^]^ Here, what must be mentioned was that in procedure V, we need to process a double spin coating to achieve high diffraction efficiency and uniform film simultaneously, in which the detailed discussion narrated in Section S8 (Supporting Information). The detailed parameters of preparation are listed in **Table** **1**.

**Table 1:** Detailed procedures during the p‐LCDG preparation.

Procedure	Process	Material	Time
I	Substrate preparing	500 µm bare glass	
II	Spin coating	SD1 (DMF:1.5wt%)	5s/800rpm + 30s/3000rpm
III	1^st^ exposure	UV light (λ = 365 nm)	60s
IV	2^nd^ exposure	UV light (λ = 365 nm) + DG mask	100s
V	Double spin coating	LCP	30s/3000rpm
VI	Ultraviolet (UV) curing	UV light (365 nm)	120s

The p‐LCDG's performance was characterized by preparation. The scanning electron microscope (SEM) structure in the diffraction state of the 7 × 7 p‐LCDG is shown in Figure 4b. The binary phase domain was identifiable. In the experimental test, the 7 × 7 diffraction lattice has a uniform distribution, as shown in Figure 4c. Most parts of the light spot have similar brightness, and certain edge spots are slightly weaker. The main reason was that the alignment direction of SD1 or LCP was not completely consistent, which reduces the diffraction efficiency. Here, the thickness of the LCP should be satisfied with the half‐wave condition. Subsequently, the brightness uniformity of the diffraction lattice is analyzed in Figure 4d. It can be seen that the brightness of light spots in the central area was significantly greater than the other spots, mainly contributing to the fabrication errors of DG. Based on the diffraction intensity data obtained in Figure 4d, we finally calculate the corresponding diffraction efficiency of 47.2% and lattice uniformity of 72.4%, in which the calculation method and measure experiment can be seen in Section S9 (Supporting Information).

### System Verification

**Figure 5:**
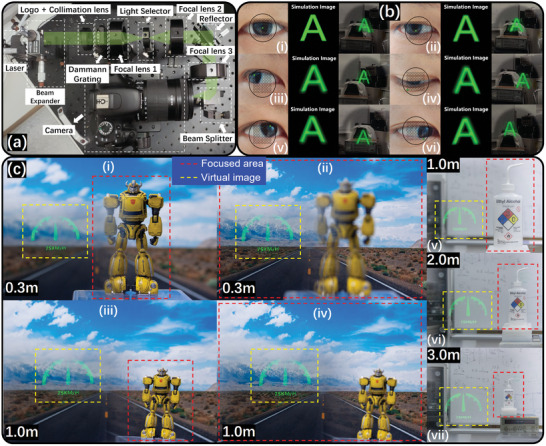
The optical verification for the proposed AR RPD system. Figure a) shows the overview of the optical layout, and the corresponding display effect in paragraphs b) shows the simulation and measured images from different positions of the viewpoint, including i) center, ii) right decentered, iii) upper right, iv) lower right, v) upper left, and vi) lower left. (Here, informed consent of the participating subject was obtained.) The VAC effect testing shown in figure c) depicts the steadily clear virtual image when the camera is focused on: 1) two objects (natural background and yellow robot), here (i) and (iii) are focused on the yellow robot that has a distance from the camera of 0.3 and 1.0 m, respectively, while (ii, iv) are focused on the natural background; 2) a moving object of a medicine bottle at the distances from the camera of v) 1.0 m, vi) 2.0 m, and vii) 3.0 m, respectively.

The experimental layout of the AR RPD system is shown in **Figure** **5**a. A monochromatic green laser (λ = 532 nm) and a light modulator with pattern templates are used for producing the virtual image. To verify the display effect, a camera was set on the image plane capturing the virtual image. The display effect of each viewpoint is shown in Figure 5b, including both simulation and experiments, respectively. It can be seen from the figures that the brightness of the central viewpoint was relatively high, exhibiting high diffraction efficiency. The brightnesses of the edge viewpoint are slightly inferior to the central position, but they demonstrate high‐intensity consistency, in which the imaging discipline is also in good agreement with the simulation results in Figure 3. Here, the switching process of our proposed RPD is shown in Video S1 (Supporting Information), and further, the comparisons with the traditional type with image missing or overlap issues are shown in Videos S2 and S3 (Supporting Information), respectively. The most important finding the figure provides was that the virtual image always appears in front of the pupil when the human eyes rotate, which realizes the functions and characteristics we desire to achieve. In addition to this, we also verify VAC free in RPD, as Figure 5c shows. For Figure 5c‐i–iv, the focus is switched between the background image and the yellow robot with two different distances of 0.3 and 1.0 m, and the virtual image maintains clarity from beginning to end, proving that the proposed AR RPD can keep the virtual image out of influence when the viewer switches the focus of eyes between two objects. For Figure 5c‐v–vii, the medicine bottle is positioned far away from the camera, representing the situation of a moving object. When continuously moving from 1.0 to 3.0 m, the virtual image remains clear, also proving the high robustness of the focus range in the proposed AR RPD. The above two situations reflect that the viewer using the proposed AR device can always focus on two different objects or a moving object freely (covering the practical usage scenarios in most cases) with a clear virtual image disregarding of VAC effect.

## 5 Conclusion

In summary, this paper effectively validates an AR RPD system with 7 × 7 switchable ultra‐dense viewpoints, utilizing a p‐LCDG to perfectly address the challenge of dynamic aperture size and pupil location changing in AR daily use. This issue often results in image loss or overlap within existing multi‐viewpoint RPD systems. Through the use of a light selector in the 4f system, the multi‐viewpoint closest to the pupil can be selected, ensuring that only one viewpoint receives the light intensity at any given time. This mechanism enables the virtual image to accurately track the pupil even as the eyes rotate. The distance between two multi‐viewpoints is reduced to 0.532 mm, which has a record high rotation precision of 1.28°/viewpoint when the eyes are rotating, much smaller than the minimum pupil radius of 1 mm for a normal person. Simulation results show a maximum GR of 19.72 µm on the focal plane of the light selector and a minimum MTF curve higher than 0.2 at the furthest area at a 45° direction on the image plane, both of which exceed the image quality demands of AR display. The experimental results demonstrate that, during the switching of multi‐viewpoints, only one virtual image consistently tracks the pupil based on eye‐tracking. This feature effectively mitigates issues related to image overlap and discontinuity in multi‐viewpoint RPD, ensuring clear imagery on the retina. Besides, the VAC effect testing also verifies that the proposed AR device can keep people seeing a steady virtual image no matter when they focus on which object in the real world without the VAC effect. Such a system enables individuals to wear AR devices at any time, disregarding the impacts of eye rotation and variations in pupil aperture. The proposed case can provide a practical solution for eye tracking type RPD in AR display, which has solved a long‐standing problem of image overlap or discontinuity caused by dynamic eye pupil.

## Conflict of Interest

The authors declare no conflict of interest.

## Supporting information



Supporting Information

Supplemental Video1

Supplemental Video2

Supplemental Video3

## Data Availability

The data that support the findings of this study are available from the corresponding author upon reasonable request.
