# Supplementary material for: Pupil‐Adaptive Retina Projection Augment Reality Displays With Switchable Ultra‐Dense Viewpoints
Source: Adv Sci (Weinh). 2025 Mar 17;12(18):2416961. doi: 10.1002/advs.202416961 (PMC12079549; doi:10.1002/advs.202416961)
Supplement: Supplementary file 1 — Supporting Information [file ADVS-12-2416961-s003.docx]

Supporting Information

Pupil-adaptive retina projection augment reality displays with switchable ultra-dense viewpoints

*Haonan Jiang^1,2^, Yuechu Cheng^2^, Zhibo Sun^2^, Zhengnan Yuan^2^, Huajian Jin^1^, Yipeng Huo^2^, Man-Chun Tseng^2^, Fion Yeung^2^, Hoi-Sing Kwok^2^, and Enguo Chen^1,2*^*

**Affiliations:**

^1^National & Local United Engineering Laboratory of Flat Panel Display Technology, Fuzhou University, 2 Xueyuan Road, Fuzhou 350108, Fujian Province, China

^2^State Key Laboratory of Advanced Displays and Optoelectronics Technologies, and Center for Display Research, Department of Electronic and Computer Engineering, Hong Kong University of Science and Technology, Clear Water Bay, Kowloon, Hong Kong, China

***Corresponding author:** ceg@fzu.edu.cn

**Part 1. The differences between pupil steering and duplication**

The method of pupil steering primarily involves changing the position of the focal plane, including moving the modulator and altering the position of light modulation on the modulator, as depicted in **Figure** **S1(a)**. The first method typically involves continuously moving a Holographic Optical Element(HOE) to change the viewpoint's position,^[S1]^ but this system is complex and difficult to compress. The second method changes the position of the light itself. With an LED array,^[S2]^ different positions of the light source can produce various viewpoints and achieve 2D eye expansion, but the resolution is not optimal. A multi-layer cholesteric liquid crystal holographic lens(CLCHL) provides high-quality images, and the focal direction changes perpendicular to the human eye, realizing foveated imaging for the retina.^[S3-S4]^ However, this method only achieves 1D eye expansion, which cannot provide enough multi-viewpoints to satisfy the free rotation of the human eye, and its structure is relatively complex.

Compared to pupil steering, pupil duplication directly copies multi-viewpoints in front of the human eye, as shown in **Figure** **S1(b)**. Multiple beams exposed on the HOE can produce multiple focuses on the pupil plane, where the resolution is considerable.^[S5]^ In the geometric method, a 2D multiple beam splitter can divide the light into nine pieces to achieve 2D eyebox expansion, but the volume is relatively large.^[S6]^ After two Pancharatnam-Berry deflectors(PBD) with a cholesteric liquid crystal(CLC) diffract the light twice, a 2D diffraction lattice is formed directly, creating 3×3 viewpoints.^[S7]^


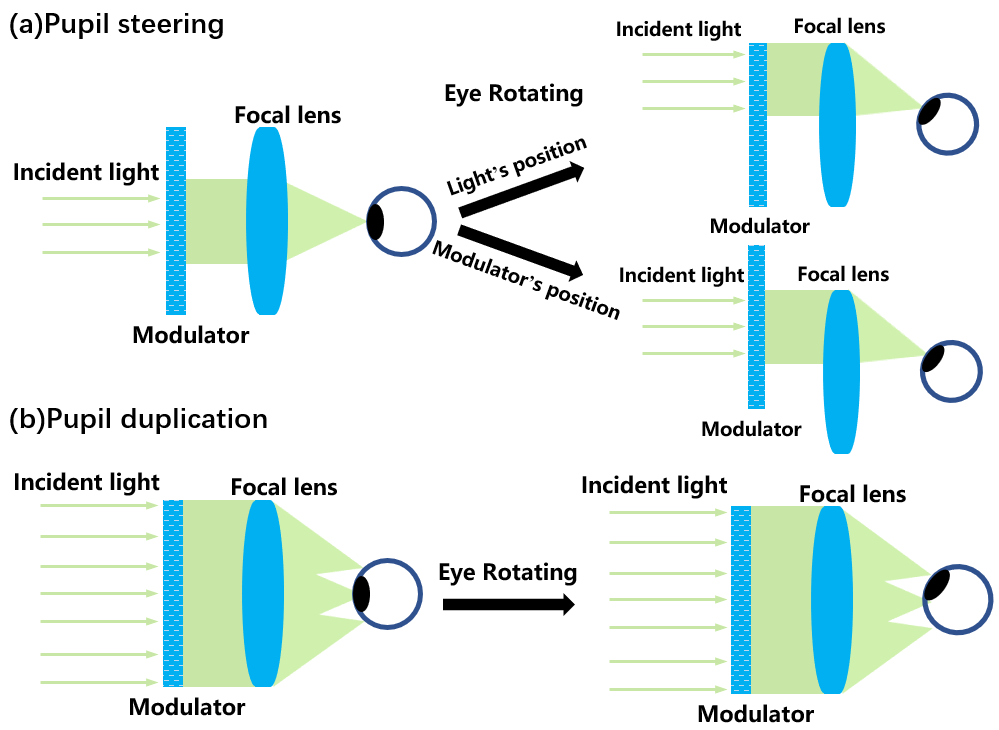


**Fig. S1** The Schematics of (a) pupil steering and (b) pupil duplication in Maxwellian display, in which the pupil steering has two methods to change the positions of the focal plane, including changing the positions of the light and modulator.

**Part 2. The effect on pupil vision with different diameters of pupil and different positions of viewpoint**

As we know, human pupils can be dynamic under different surrounding lights, especially outdoor conditions during the day and night. **Fig.** **S2** shows the effect of pupil changing on the distance between two diffraction spots. When the diameter of the pupils is 2 mm, we can find in **Fig. S2(a)** that the eye model cannot receive the virtual image when it is located at the center of the interval between two viewpoints, causing the image missing. When the pupil’s diameter changes to 4 mm in **Fig.** **S2(b)**, the eyebox increases because of the extension of the viewing range. By comparison among **Figs.** **S2(a)(b)** and **(c)(d)**, the change of spot distance can not only affect the eyebox but also rotation precision. In this way, it is essential to make a balance between spot distance and rotation precision to make sure that the human pupil can adapt to the surrounding light changing when using AR devices outside.


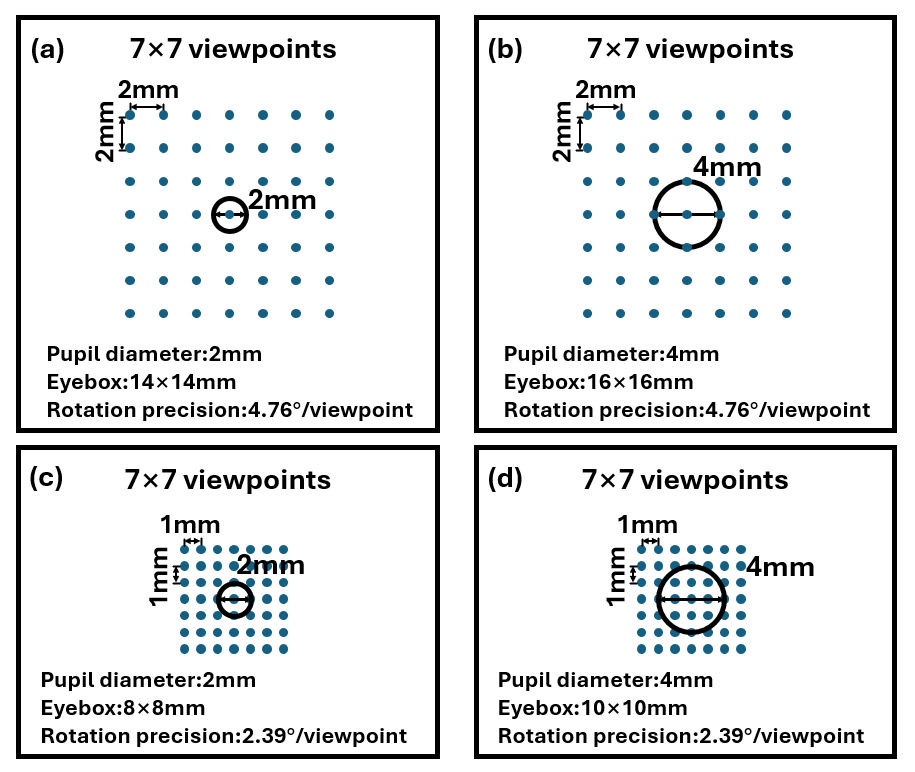


**Fig.** **S2** The relationship between different pupil diameters and viewpoint distance.

**Part 3. Optical parameters of the commercial lens surface**

To keep the feasibility of the proposed system, we choose a commercial 10th-order aspherical lens with high imaging quality settled in the experiment. Its detailed datasheet is presented in **Table** **S1**. The overview of the 3D model is shown in **Figure** **S3(a)**, which includes half FOV from 0° to 3°. Here, considering the diffraction caused by p-LCDG, we need to analyze the imaging quality covering half FOV from 0° to 3°, including both the RMS spot and wavefront map(depicted in **Figures** **S3(b)** and **(c)**). Also, in MTF performance in **Figure** **S3(d)**, we find that half of FOV within 3° all show a resolution >0.2@62.5lp/mm, which is high enough to meet with the off-axis imaging quality caused by diffraction.

**Table S1.** Detailed optical parameters of the aspherical lens from *Edmund optics*.

| Surface Type | | Radius | Thickness | Material | Coating | Clear Semi-Dia | Conic | 4th Order | 6th Order | 8th Order | 10th Order |
| --- | --- | --- | --- | --- | --- | --- | --- | --- | --- | --- | --- |
| **OBJ** | Standard | Infinity | Infinity |  |  |  |  |  |  |  |  |
| STOP (aper) | Even Asphere | 40.259 | 13.058 | N-SF6 | EO_VISEXT_785 | 22.500 | -0.770 | 1.479  E-07 | -7.468  E-11 | -5.681  E-14 | 1.783  E-17 |
| (aper) | Standard | Infinity | 42.768 |  |  | 22.500 |  |  |  |  |  |
| **IMA** | Standard | Infinity |  |  |  |  |  |  |  |  |  |


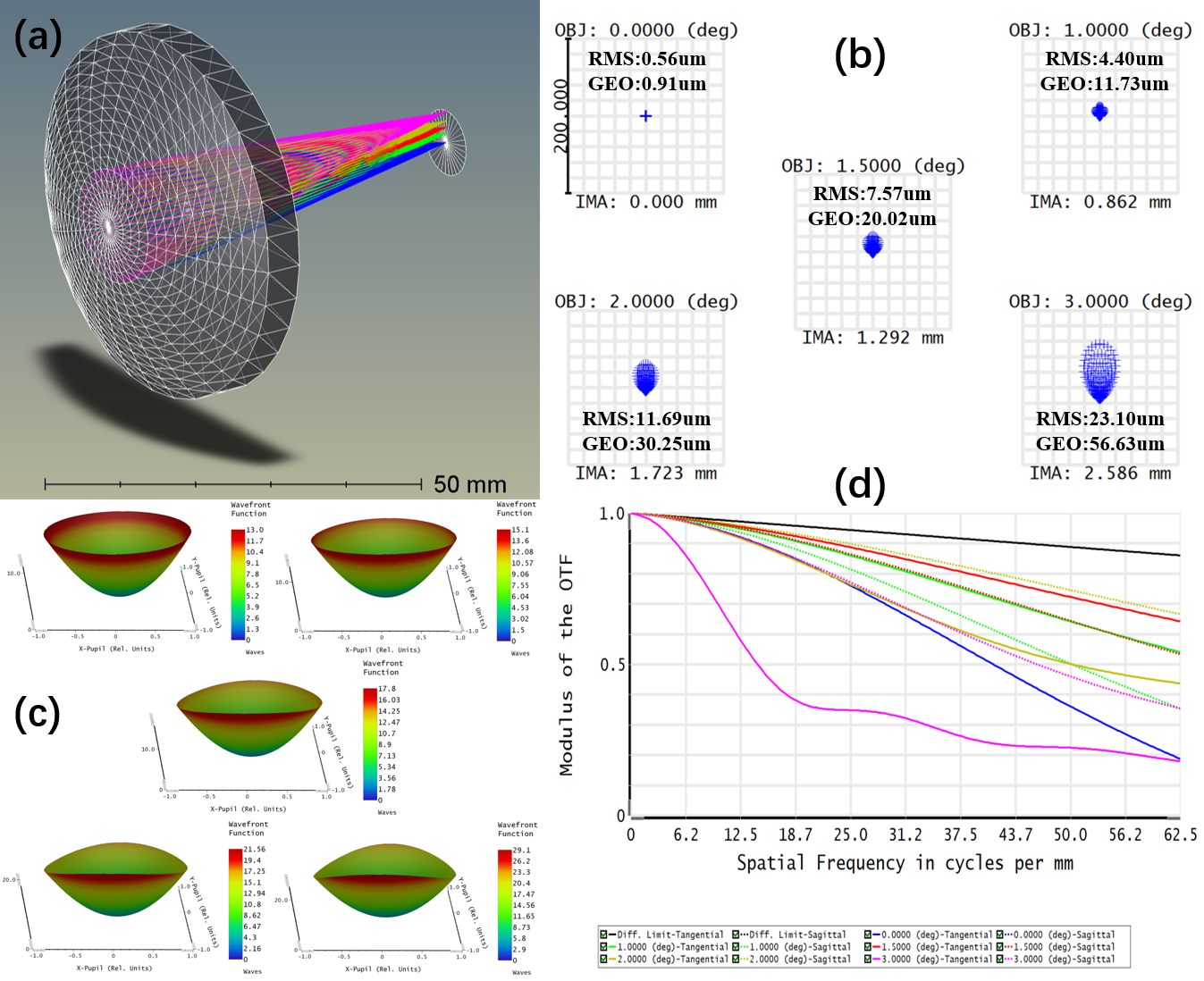


**Fig. S3** The imaging quality analysis for commercial lens from *Edmund optics* induced by simulation. (a) The 3D overview for the lens. (b) The RMS spot from half FOV of 0° to 3°. (c) The corresponding wavefront error is visualized by the 2D diagram. (d) MTF performance both in tangential and sagittal direction with diffraction limit.

**Part 4. The 10 viewpoints’ analysis of the GEO radius on the focal plane of the light selector and the MTF performance on the image plane of the system**

To conduct comprehensive research, we analyze the GEO radius on the light selector's focal plane and the MTF performance on the image plane in detail(see **Figures** **S4** and **S5(a)-(j)**). Here, we select 10 viewpoints based on the symmetry of the viewpoints, along the directions of horizontal, vertical, and 45°, respectively. From **Figures** **S4** and **S5(a)**, we can find that the spot and MTF in the central viewpoint are nearly perfect, almost equal to the diffraction limit, which also highlights the advantage of foveated imaging in RPD. However, the performances on the off-axis viewpoints (**Figures** **S4** and **S5(b)-(j)**) deteriorate sharply from center to edge, especially in the MTF curve. From the spot diagrams at the 45° direction (**Figures** **S4(h)-(j)**), we see the main geometric aberration is wisdom difference, which is caused by the system's aperture. However, there is a balance between the aperture and the FOV, so the aperture cannot be too narrow. The MTF on the directions of horizontal and vertical (**Figures** **S5(b)-(g)**) show distinct differences in meridional and sagittal planes, which are mainly caused by the limited phase precision at x and y directions in the commercial aspherical lens, but anyway the MTF performances at all viewpoints are higher than 0.2@62.5lp/mm, which meet with the basic resolution requirement in AR display. According to the analysis above, the aberration correction produced by the Dammann grating is essential for further research.

**
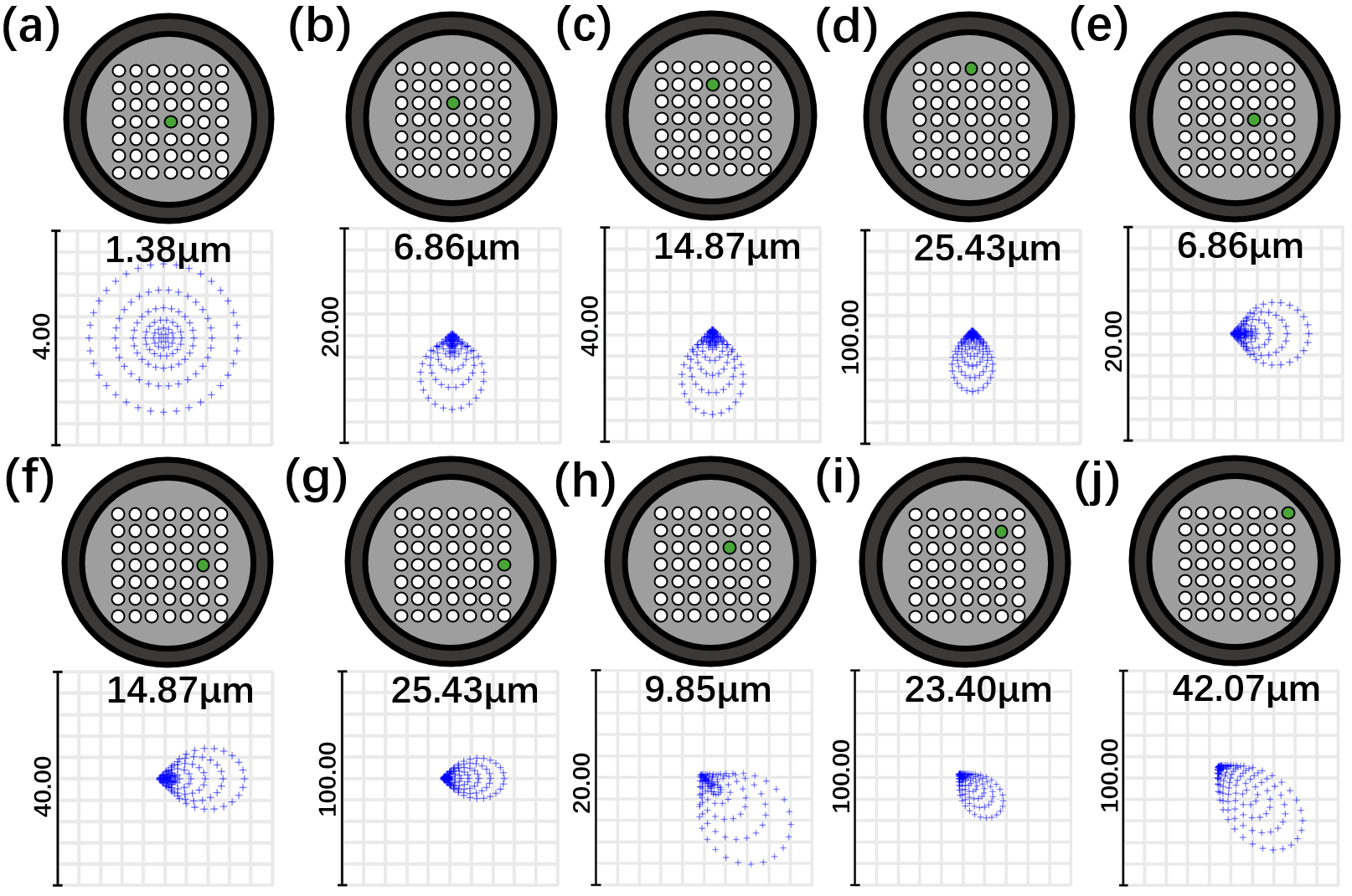
**

**Fig. S4** The GEO radius on the focal plane of the light selector. (a) is at the central viewpoint of the light selector. (b)-(d) represents the viewpoints along the vertical direction (e)-(g) represents the horizontal direction, and (h)-(j) shows the oblique direction with 45°.


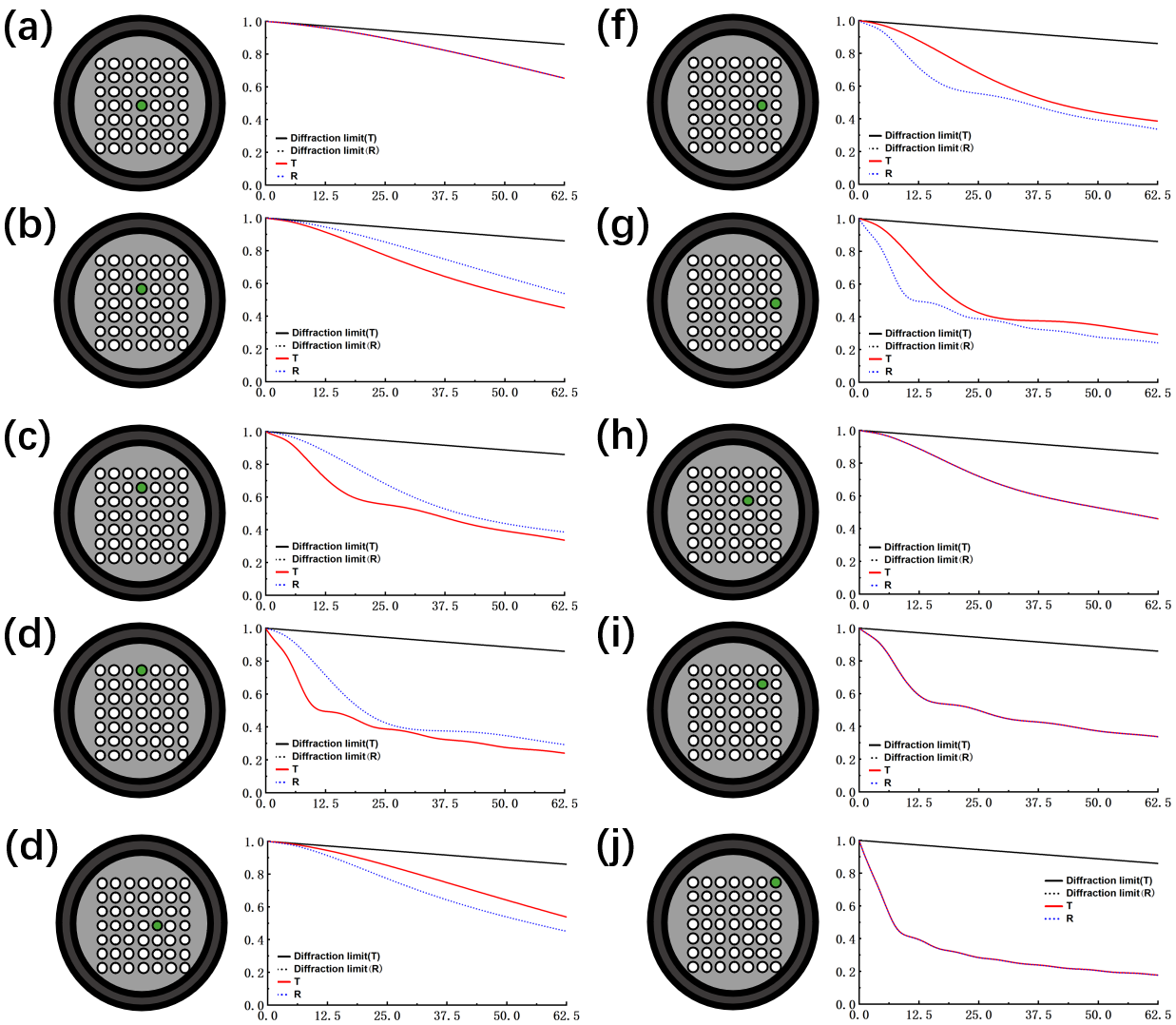


**Fig. S5** The MTF performance on the image plane of the system. (a) is at the central viewpoint of the light selector. (b)-(d) represents the viewpoints along the vertical direction (e)-(g) represents the horizontal direction, and (h)-(j) shows the oblique direction with 45°.

**Part 5. The effect on the system with different optical parameters**

In this section, we mainly explore the effect of changing some essential optical parameters on the optical performance of the proposed system, as shown in **Figure** **S6**. To control the variables, we set the grating period to 50 μm in the focal length curve and the focal length to 50mm in the grating period curve in **Figure** **S6(a)**. According to **Equations** **(1)** and following **S(1)**:

$\tan\frac{w}{2}=\frac{D}{2f}$ S(1)

where w represents the FOV and, f is defined as the focal length, and here, D is the light aperture in Maxwellian display instead of the image height.^[S6]^ As can be seen in the figure, when the focal length of the lens increases, the distance of the light spot also increases. However, when the grating period increases, the spot distance decreases. Therefore, we need to consider these factors comprehensively when setting these three parameters. In general, a short focal length and a large grating period can make both the volume and image quality relatively good but also lead to a narrow distance between diffraction light spots, causing a small eye box simultaneously.

Subsequently, we explore the relationships between the light aperture and FOV/MTF performance at the central viewpoint, as shown in **Figure** **S6(b)**. From the curve, we find that as the light aperture increases, both the FOV and the MTF on the image plane increase. Here, the principle of FOV changing can be explained by **Equation (2)**. Moreover, since the light is incident parallel to the system, the entrance pupil diameter(EPD) is equal to the light aperture. If the focal length remains unchanged, the increase of the EPD will cause a decrease in the F-number. As a result, the diameter of the Airy disk decreases, and the minimum spatial resolution also decreases, which further leads to a rise in the image quality in the cut-off frequency of MTF when the pixel size is fixed. It can be seen that the MTF and FOV at the central viewpoint can be improved by expanding the light aperture, but we still need to consider the effect of the increasing volume of the system.

As is known, the position of the edge viewpoint directly determines the overall range of the diffraction lattice, which also measures the eyebox, and the grating period indirectly determines the diffraction spot distance. These factors all affect the MTF performance at the edge viewpoint, so we analyze the impact of the grating period on MTF performance and eyebox at the edge viewpoint. According to the curve shown in **Figure** **S6(c)**, the increase in the grating period leads to a decrease in the eyebox but improves the MTF performance. Therefore, a trade-off needs to be made between image quality and eyebox, and it is certain that the MTF performance at the edge viewpoint also depends on the design of the focal lens.


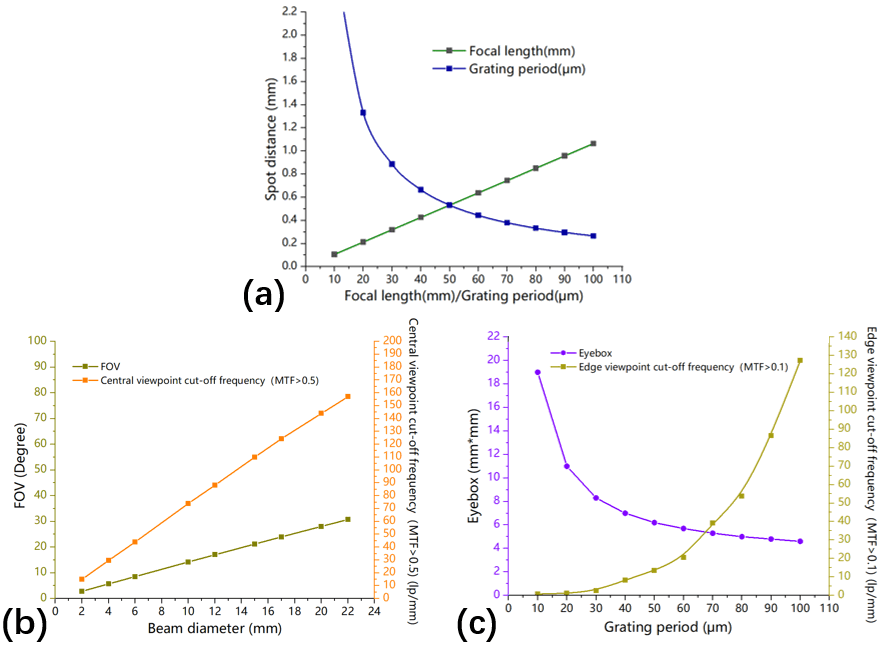


**Fig. S6** The effect on the optical performance of the system when some essential parameters are changed. (a) The relationships between the spot distance of Dammann grating and the grating period and focal length. (b)The relationships between the light aperture and FOV/MTF performance at the central viewpoint. (c) The relationships between the grating period and the eyebox/the MTF performance at the edge viewpoint.

**Part 6. The calculation about the size of the eyebox.**

Commonly, traditional AR/VR devices calculate the eyebox by measuring the size of the virtual image and the distance between the pupil and the image. Unlike that, RPD usually measures its viewpoint range together with the human vision range, as shown in **Fig. S7**. In this paper, we set pupil diameter as the average value of 3 mm, so the whole range of eyebox is calculated to be 0.532×6+1.5×2, equal to 6.192 mm.


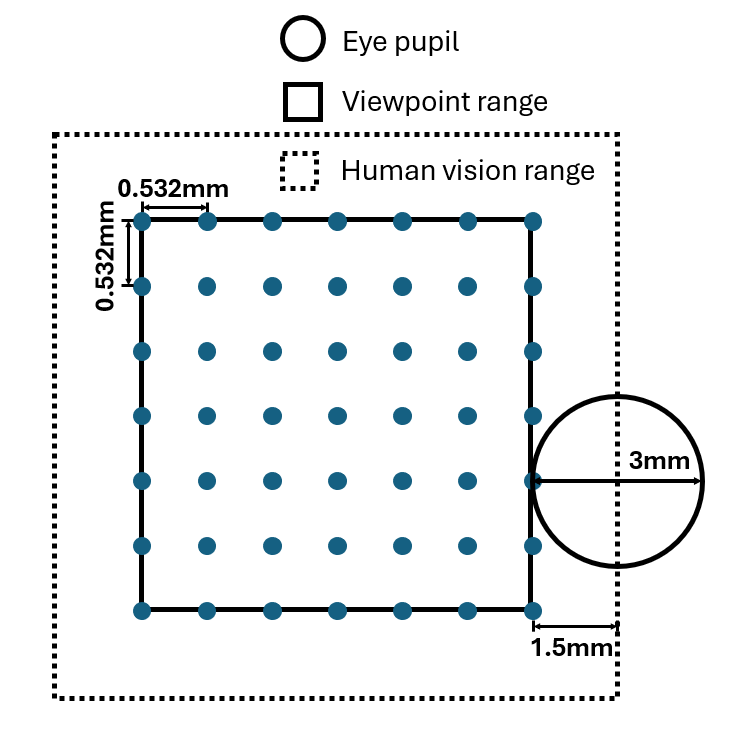


**Fig.** **S7** The schematic diagram of eyebox calculation.

**Part 7. The derivation process of 7×7 DG mask**

In Dammann gratings, the multiple dispersion of light is achieved through interference phenomena. When incident light hits the grating, it is dispersed into different wavelengths. Consequently, light with different wavelengths produces distinct interference fringes and forms various interference angles. These interference angles affect the direction of light, causing light with different wavelengths to disperse in separate directions. In modern Dammann grating design, we typically create a one-dimensional structure with specific phase change point coordinates within one period to achieve uniform light intensity in the diffraction lattice. Afterward, we can repeat the structure of one period and expand it along the orthogonal direction into a two-dimensional structure of any desired size. Finally, we obtain the two-dimensional Dammann grating.

In this study, we use a Dammann grating with a 7×7 diffraction lattice. According to previous research that calculates the optimal phase change points from a 1×1 to 64×64 diffraction lattice,^[S8]^ we can obtain the coordinates of phase change points for a 7×7 Dammann grating as x=0.23191, 0.42520, and 0.52571. The phase distribution is shown in **Figure** **S8(a)**. Thus, we expand it into a two-dimensional structure orthogonally and obtain the phase distribution of the normalized period, which is illustrated in **Figure** **S8(b)**. By using **Equation** **S(2)**, we can determine the required grating period:

$\sin\theta=\frac{\lambda}{P}$ S(2)

where θ represents the diffraction angle, λ denotes the wavelength of the incident light, and P signifies the grating period.


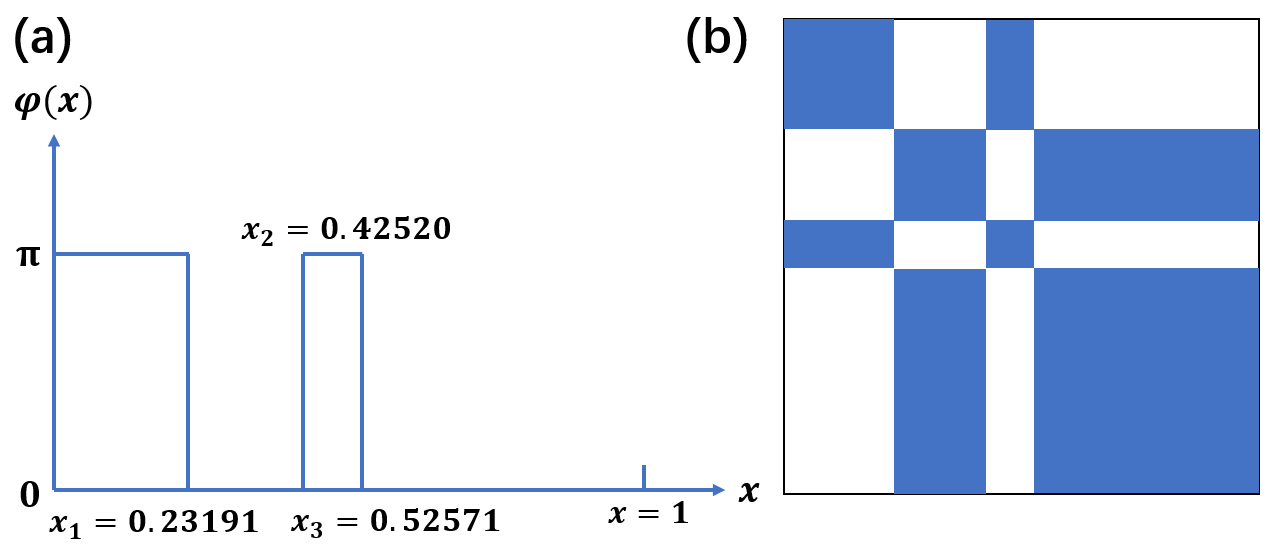


**Fig. S8** (a) The 1D phase distribution of 7*7 Dammann grating for the normalized period. (b) The corresponding 2D phase distribution for the normalized period after orthogonal expanding.

**Part 8. The** **Effects of LCP concentration and spin coating speed on the thickness of the LC layer**

As we all know, the LC element can achieve the highest efficiency only when its thickness satisfies the half-wave conditions given by **Equation** **S(3)**:

$d=\frac{\lambda}{2\Delta n}$ S(3)

where d represents the thickness of the LCP layer, λ denotes the wavelength of the incident light and signifies the birefringence of LCP. However, the film thickness processed by spin coating is determined by various factors, such as solution concentration and rotating speed. To find an optimal spin coating scheme, we measure the LCP thickness with different concentrations and rotating speeds listed in **Table** **S2**. Finally, according to the designed light wavelength and specified LCP material, double spin coating with 20% LCP and 3000rpm(30s) is the best.

**Table S2.** LCP thickness(nm) measurement with different concentrations and spin coating speeds

| Concentration(percent)  Spin Coating Speed(rpm) | 10%LCP | 20%LCP | 30%LCP |
| --- | --- | --- | --- |
| 1000 | 1127 | 1372 | 1546 |
| 2000 | 775 | 941 | 1079 |
| 3000 | 615 | 786 | 920 |
| 4000 | 572 | 709 | 778 |

**Part 9. The measurement of diffraction efficiency and uniformity calculation for Dammann grating**

In the experiment of diffraction efficiency measurement for Dammann grating, we usually test the light intensity for every single spot by observing the photovoltage provided by the photodetector. The corresponding experimental device setting is shown in the following **Fig.** **S9**. Here, due to the fixed load resistance, we can straightly use the obtained photovoltage to obtain the light intensity for each spot. In this experiment, we obtain the 49-photovoltage for 7×7 Dammann grating, shown in **Fig** **S9**. After obtaining the photovoltages, we use them as the absolute value of light intensity. Here, we use some ***Statistical analysis*** as follows:

In the calculation of diffraction efficiency for Dammann grating, we obtain the light efficiency by getting the ratio between output diffracted light intensity and input total light intensity, which is followed by this formula:

$$\eta=\frac{I_{0}+2\sum_{\text{i}=1}^{N} I_{i}}{I_{total}}\times100\%$$

where *η* is the light efficiency, *I*_0_ is zero-order diffraction light, N is equal to the highest diffraction order, and *I*_total_ represents the total input light intensity. Based on the experiment results in **Fig.** **S10**, we obtain output diffracted light intensity *I*_output_=2087.88mV and the *I*_total_=4.42V, in which the light efficiency is equal to 47.2%. When we discuss the reason why this result is significantly lower than the simulated theoretical limit value(≈61.8%^[S8]^), we consider that it can contribute to the following three reasons: 1) The surrounding light’s crosstalk; 2) The fabrication error caused by the uncomplete alignment of SD1 and LCP layer; 3) The thickness error in LCP layer.

In the calculation of uniformity for Dammann grating, we discuss it as the same as that in the display panel, which is intuitive in AR display. The corresponding value can be obtained by the following formula:

$$Uni=\frac{\mathrm{I}_{\min}}{I_{\max}}\times100\%$$

where *I*_min_ and *I*_max_ represent the minimum and maximum light intensity among all the diffraction light in Dammann grating, in this paper, from **Fig.** **10,** we can get the minimum and maximum values of 37.9mV and 52.37mV respectively, and the corresponding uniformity is equal to 72.4%.


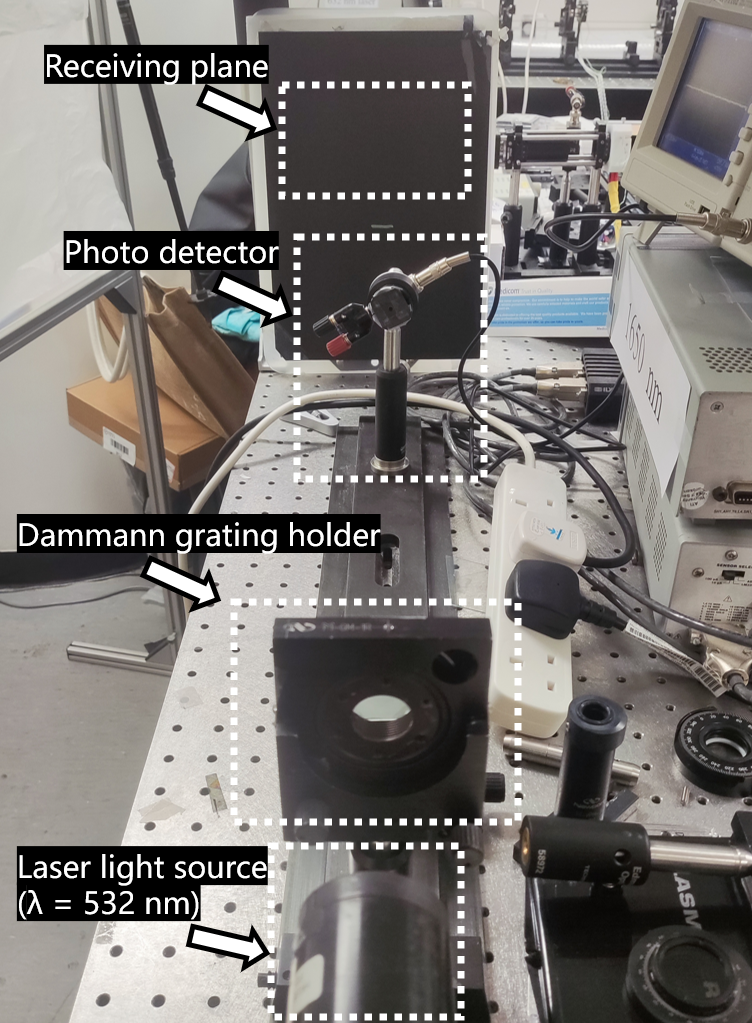


**Fig. S9** The schematic diagram of light intensity measurement.


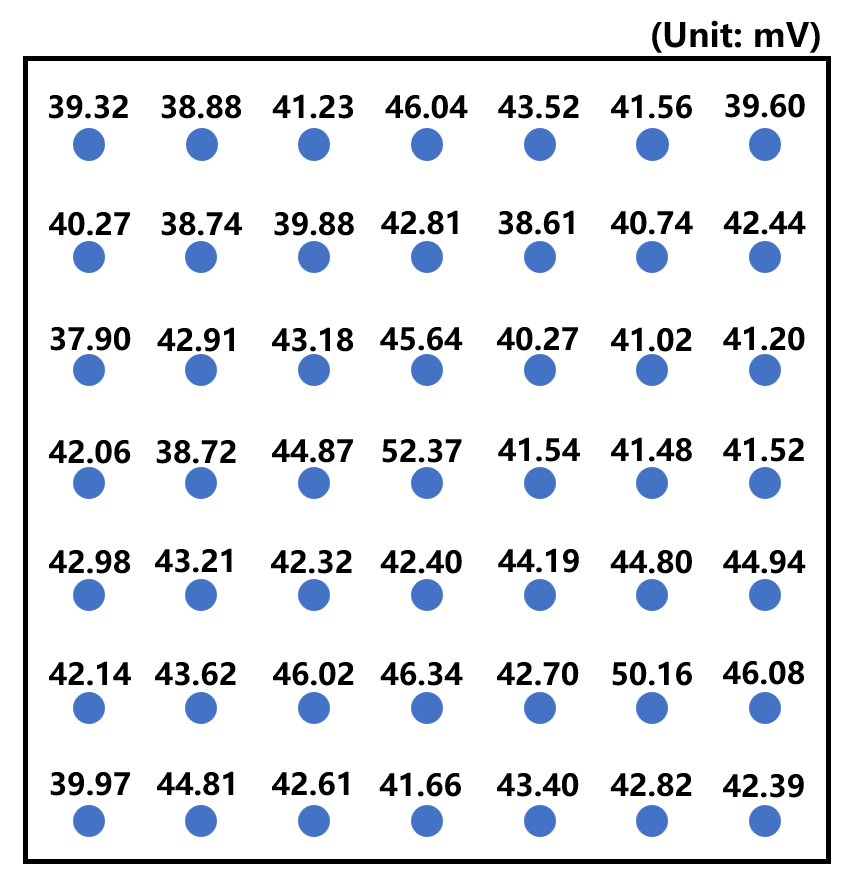


**Fig. S10** The photovoltage of every single diffraction spot.

**References**

1. J. Kim, Y. Jeong, M. Stengel, K. Akşit, R. Albert, B. Boudaoud, T. Greer, J. Kim, W. Lopes, Z. Majercik, P. Shirley, J. Spjut, M. Mcguire, D. Luebke *ACM Trans. Graph.* **2019**, *38*, 99.
2. T. Ueno, Y. Takaki *Opt. Express* **2018**, *26*, 30703-30715.
3. J. Xiong, Y. Li, K. Li, S.-T. Wu *Opt. Lett.* **2021**, *46*, 1760-1763.
4. J. Zou, L. Li, S.-T. Wu *Adv. Photon. Res.* **2022**, *3*, 2100362.
5. S.-B. Kim, J.-H. Park *Opt. Lett.* **2018**, *43*, 767-770.
6. P. K. Shrestha, M. J. Pryn, J. Jia, J.-S. Chen, H. N. Fructuoso, A. Boev, Q. Zhang, D. Chu *Research* **2019**, 9273723.
7. T. Lin, T. Zhan, J. Zou, F. Fan, S.-T. Wu *Opt. Express* **2020**, *28*, 38616-38625.
8. C. Zhou, L. Liu *Appl. Optics* **1995**, *34*, 5961-9.
